# The Effect of Ultrasonic Cleaning on the Secondary Electron Yield, Surface Topography, and Surface Chemistry of Laser Treated Aluminum Alloy

**DOI:** 10.3390/ma13020296

**Published:** 2020-01-09

**Authors:** Jie Wang, Yong Gao, Zhiming You, Jiakun Fan, Jing Zhang, Sheng Wang, Zhanglian Xu

**Affiliations:** Shaanxi Engineering Research Center of Advanced Nuclear Energy & Shaanxi Key Laboratory of Advanced Nuclear Energy and Technology & School of Nuclear Science and Technology & School of Energy and Power Engineering, Xi’an Jiaotong University, Xi’an 710049, Shaanxi, China; wangjie1@xjtu.edu.cn (J.W.);

**Keywords:** laser ablation, surface engineering, materials processing, surface morphology, aluminum alloy

## Abstract

Laser ablation technique is a novel method for obtaining a surface with a low secondary electron yield (SEY) that can mitigate electron cloud in high-energy accelerators. Before the installation of laser processed aluminum alloy, surface cleaning is of the essence to reduce the contaminations of ultra-high vacuum systems for providing appropriate pressure for beam operation consequently. Laser processed aluminum alloy is one of the crucial candidates for the vacuum system construction of future accelerators. Moreover, ultrasonic cleaning is an essential procedure for most materials applied in vacuum systems. Therefore, in order to verify the stability of the laser created structures by ultrasonic cleaning and evaluate the impact of the cleaning on the SEYs, the surface topographies, and the surface chemistries of laser treated aluminum alloy, SEY measurements and related tests were performed. After ultrasonic cleaning, the SEYs of laser treated aluminum alloy increased from 0.99, 1.05, and 1.16 to 1.43, 1.74, and 1.38, respectively. Compared to the surface roughness of uncleaned laser treated aluminum samples, the cleaned laser treated ones decreased from 10.7, 7.5, and 14.5 to 9.4, 6.9, and 12.9, respectively. The results indicate that ultrasonic cleaning can induce the SEY increase of laser processed aluminum alloy. The correlative mechanism between the surface morphology, the surface chemistry, and SEY increase were analyzed for the first time.

## 1. Introduction

Surface contamination, which can affect the beam quality and beam lifetime, is undesirable for the ultra-high vacuum (UHV) system of particle accelerators [1,2,3,4,5]. Dynamic degassing caused by synchrotron radiation and particles bombardment will influence the beam lifetime and even cause beam breakup in particle accelerators [6,7]. In order to reduce the contaminants, the components in the vacuum system need to be cleaned according to the regulated cleaning procedures [8,9]. Chemical cleaning is one of the common methods for the removal of surface contaminants in UHV systems [10]. A variety of solvents, such as perchloroethylene, acetone, absolute ethyl alcohol, etc., are usually adopted for the cleaning of vacuum products made of stainless steel, copper, and aluminum alloy [11,12,13]. Ultrasonic cleaning combined with appropriate solvents is usually used for surface cleaning of different UHV components in accelerators, such as thin film coatings [14,15], SRF cavities [16,17], laser engineered surfaces [18], high-heat-load absorbers [19], etc.

Laser processed aluminum alloy was proposed by Wang et al. [20] as a prospective option for electron cloud (EC) mitigation, which could be used in the vacuum system of accelerators. The related properties of laser treated metals for EC inhibition were investigated for the past few years, such as the surface resistance of laser treated copper samples [21], the adsorption and desorption properties of laser processed copper [22], and the empirical model of laser treated porous stainless steel surface with gold film coatings [23]. These research results provide valuable information on the properties of laser treated metals, for example, laser treated copper plates and stainless steel sheets. 

While surface contaminations, such as powders formed on the laser treated surface after laser processing, should be cleaned carefully before its installation in the vacuum system of the next-generation accelerators. The residual powders on laser treated aluminum surface could be radiated and bombarded by synchrotron radiation and lost beam particles, resulting in the interaction with beam particles and affecting the beam quality and beam lifetime ultimately. In order to meet the requirement of ultrahigh vacuum in accelerators, the laser processed aluminum alloy surface should be cleaned. The removal of adsorbed or residual contaminants and particles is much more effective by ultrasonic cleaning [4]. The ultrasonic cleaning procedures include the cleanings in various kinds of solvents in a certain period of time [4]. However, the mechanism of an ultrasonic cleaning effect on surface morphologies, surface chemistry, and secondary electron yield of laser treated surface is not clear yet and should be investigated systematically. 

To verify the stability of the laser created structures, such as floccule/fiber-like structures, by ultrasonic cleaning and investigate the impact of the cleaning on the secondary electron yields (SEYs) of laser processed aluminum alloy, the relevant experiments and analyses were conducted. Moreover, many factors, such as surface compositions, surface roughness, etc., can affect the SEY results. Therefore, the relation between the surface chemical states, surface morphologies, and the SEY variation of laser processed aluminum alloy samples before and after ultrasonic cleaning were studied for the first time in this paper.

## 2. Experiments and Methods 

### 2.1. Sample Preparation and Cleaning Procedures

**Figure 1:**
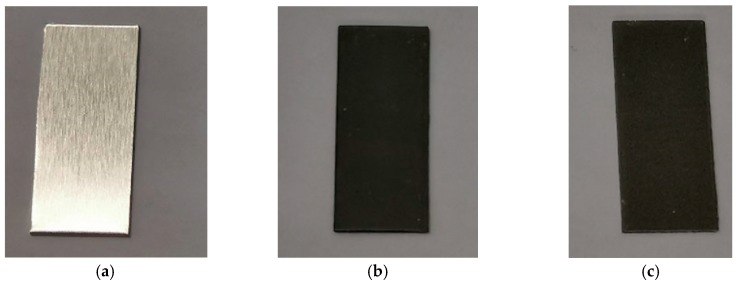
The real photos of (**a**) the untreated aluminum alloy, (**b**) the laser treated aluminum alloy, and (**c**) the cleaned and laser treated aluminum alloy samples.

The 6061-T6 aluminum alloy samples were processed by laser in air. The chemical compositions of 6061-T6 aluminum alloy were Al element of 97.961% and other elements (such as Mg, Si, Fe, and Cu) of 2.039%. The real photos of the untreated aluminum alloy, the laser treated aluminum alloy, and the cleaned and laser treated aluminum alloy samples are shown in Figure 1. 

In order to remove the powders formed on the laser treated surface during laser processing, the laser treated samples with dimensions of 0.5 mm × 9 mm × 20 mm (for SEY tests) and 0.5 mm × 10 mm × 10 mm (for other tests) were put in a beaker of 50 ml and cleaned ultrasonically in acetone and absolute ethyl alcohol for 20 min, respectively, which is the typical time used to clean a real part in the same bath. During the cleaning, the power and the frequency of the ultrasonic cleaner (Doyes CJ-010s, Huace Science and Technology Limited company, Shenzhen, China) were 80 W and 40 kHz, with a volume of 2.0 L. The selection of the ultrasonic cleaner was mainly based on the sample size, the sample position, and the bath volume. The laser treated samples before and after cleaning were taken for related tests to evaluate the effect of ultrasonic cleaning on their surface morphologies, chemical states, and secondary electron yields.

### 2.2. Laser Parameters

The laser parameters (K20-CS nanosecond pulsed fiber laser, Han’s Laser, Shenzhen, China) and the maximum SEYs (*δ*_max_) of different laser processed aluminum alloy samples are shown in Table 1. All the aluminum alloy samples were processed by an average laser power of 13.33 W, a laser wavelength of 1064 nm, and a laser spot size of 15 μm with equidistant or non-equidistant hatched patterns. Here, the non-equidistant hatched pattern is a repetition of a sequence of separations (5 μm, 10 μm, 10 μm, and 25 μm) repeated every 50 microns. The pitch spacings of samples #1 and #2 were 15 and 20 μm, respectively, with scanning speeds of 100 and 150 mm s^−1^, respectively. The scanning speed of sample #3 was 150 mm s^−1^. Samples #1 and #2 were processed with a equidistant line hatched pattern, and sample #3 with a non-equidistant hatched pattern. The selection of the laser parameters in this study was based on the research by Wang et al. [20].

### 2.3. Characterization Method

The SEYs of laser processed aluminum samples before and after ultrasonic cleaning were investigated using the SEY testing devices introduced by Wang et al. [20]. The beam diameter is approximately 1 mm, with an electron beam current of around 10 nA. The tests of surface chemical states were carried out using AXIS ULtrabld X-ray photoelectron spectroscopy (XPS) with the operating pressure of about 10^−7^ Pa. The C 1s peak of 284.8 eV was employed for the binding energy calibration. Individual samples of the area of 10 × 10 mm^2^ were mounted on the sample holder. After laser processing, the samples were examined by a JEOL 7800F Schottky field scanning electron microscope (SEM, Tokyo, Japan) to determine the surface morphologies. The surface particle sizes and densities of different aluminum alloy samples before and after ultrasonic cleaning were obtained and analyzed based on SEM images. The surface roughness shown in Table 1 were obtained by an Olympus OLS4000 laser scanning confocal microscopy (LSCM). A Helios NanoLab 600 dual-beam focused ion beam-scanning electron microscope (FIB-SEM, FEI, Hillsboro, OR, USA) with an energy dispersive spectrometer (EDS) system was employed to characterize the cross section morphology and the elements’ distributions. The crystalline structures of cleaned and uncleaned laser treated aluminum alloy were analyzed using the PANalytical X’Pert PRO X-ray diffraction (XRD, PANalytical, almelo, Netherlands).

## 3. Results and Discussion

### 3.1. The Effect of Ultrasonic Cleaning on SEY

**Figure 2:**
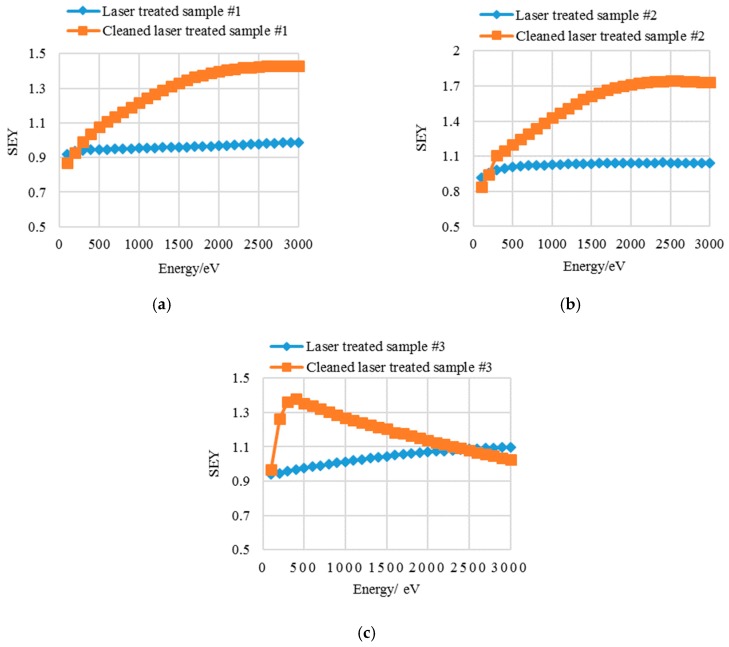
The secondary electron yield (SEY) curves of uncleaned and cleaned laser treated (**a**) samples #1, (**b**) #2, and (**c**) #3.

The SEY properties of laser processed aluminum samples with equidistant and non-equidistant hatched patterns before and after ultrasonic cleaning are shown in Figure 2. 

In Figure 2a, when the primary energy (*E*_p_) is less than 200 eV, the SEYs of cleaned laser treated sample #1 are slightly lower than those of the uncleaned one, while at 300 eV ≤ *E*_p_ ≤ 3000 eV, the SEYs of cleaned laser treated sample #1 are obviously higher than those of the uncleaned one. The maximum SEY of uncleaned and cleaned laser processed sample #1 are 0.99 and 1.43, respectively, with a corresponding energy (*E*_max_) of 3000 eV and 2700 eV, respectively.

The SEY changing trend is basically the same for sample #2 before and after ultrasonic cleaning. The *δ*_max_ of uncleaned and cleaned laser processed sample #2 are 1.04 and 1.73, respectively, with an *E*_max_ of 3000 eV and 2600 eV, respectively. Furthermore, the shape of SEY curves for uncleaned and cleaned laser treated samples #1 and #2 are leveling off as the primary energy reaches up to 3000 eV.

For sample #3, the laser hatched pattern is a non-equidistant hatched spacing. The SEYs of cleaned laser treated sample #3 are higher than those of the uncleaned one at *E*_p_ ≤ 2400 eV, as shown in Figure 2c, whereas at 2400 eV ≤ *E*_p_ ≤ 3000 eV, the SEY values of cleaned laser treated sample #3 are slightly lower than those of the uncleaned one. The *δ*_max_ of uncleaned and cleaned laser processed sample #3 are 1.10 and 1.38, respectively, with an *E*_max_ of 3000 eV and 400 eV, respectively. At the crucial energy range for electron cloud formation in accelerators [24] of *E*_p_ ≤ 500 eV, the maximum SEYs of cleaned laser treated samples #1, #2, and #3 are 1.08, 1.20, and 1.38, respectively.

Interestingly, compared to the *E*_max_ of uncleaned laser processed sample #3, that of the cleaned one decreases dramatically, from 3000 eV to 400 eV, while the *E*_max_ differences of cleaned and uncleaned samples #1 and #2 are relatively small. *E*_max_ is the energy that the maximum penetration depth of primary electrons equals the maximum escape depth of the secondary electrons [25], which can be influenced by the surface contamination and surface roughness in this study. The main difference of laser parameters for these three samples is the laser hatched pattern. The laser hatched pattern could influence the surface roughness. Thus, the significant *E*_max_ difference of uncleaned and cleaned sample #3 may be ascribed to the non-equidistant hatched spacing pattern, which is a repetition of a sequence of separations of every 50 μm (5 μm, 10 μm, 10 μm, and 25 μm). The hatched curve depths and the space between different curves of sample #3 are different, which may affect the overall maximum escape depth of secondary electrons and then influence the position of *E*_max_.

### 3.2. The Effect of Ultrasonic Cleaning on Surface Morphology

To evaluate the effect of ultrasonic cleaning on surface morphologies of laser treated aluminum alloy samples, SEM tests were performed to obtain the surface topography images. In this study, two different laser hatched patterns were adopted.

**Figure 3:**
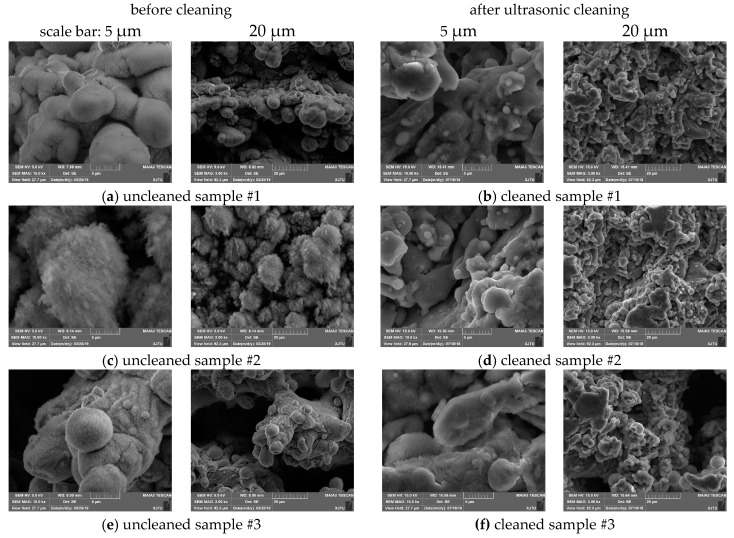
The surface SEM images of laser treated samples #1, #2, and #3. (**a**) Uncleaned laser-processed sample #1, (**b**) cleaned laser-processed sample #1, (**c**) uncleaned laser-processed sample #2, (**d**) cleaned laser-processed sample #2, (**e**) uncleaned laser-processed sample #3, and (**f**) cleaned laser-processed sample #3.

As shown in Figure 3, the average particle sizes of laser treated aluminum alloy samples #1, #2, and #3 are obviously larger than those of the ultrasonically cleaned ones. The particles with a size of 1~3 μm that appeared after ultrasonic cleaning are shown in Figure 3b,d,f, compared to those of the uncleaned ones indicated in Figure 3a,c,e. The density of floccule/fiber-like structures on the uncleaned sample surface decreased significantly. From this, it can be speculated that the floccule/fiber-like structures were mostly removed during the ultrasonic cleaning process, resulting in the decrease of average particle sizes and the appearance of much smaller particles.

**Figure 4:**
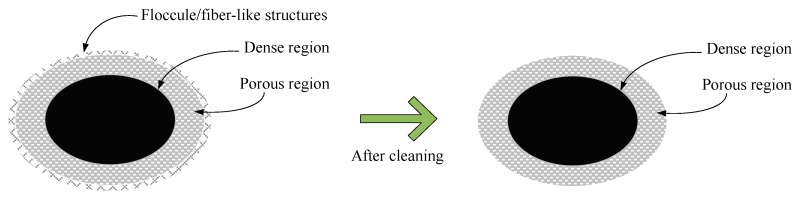
The effect mechanism of ultrasonic cleaning on surface morphology change.

As indicated in Table 1, the surface roughness of the laser processed aluminum alloy in samples #1, #2, and #3 decreased about 8–12% after ultrasonic cleaning. The surface roughness difference induced by ultrasonic cleaning may be one of the main reasons for SEY changes. The decrease of surface roughness may be related to the removal of floccule/fiber-like structures. The mechanism of the effect of ultrasonic cleaning on surface morphology change is shown in Figure 4. More precisely, the floccule/fiber-like structures were stripped out from the sphere/spheroidicity-like micron level grains during ultrasonic cleaning.

### 3.3. The Effect of Ultrasonic Cleaning on Surface Composition

**Figure 5:**
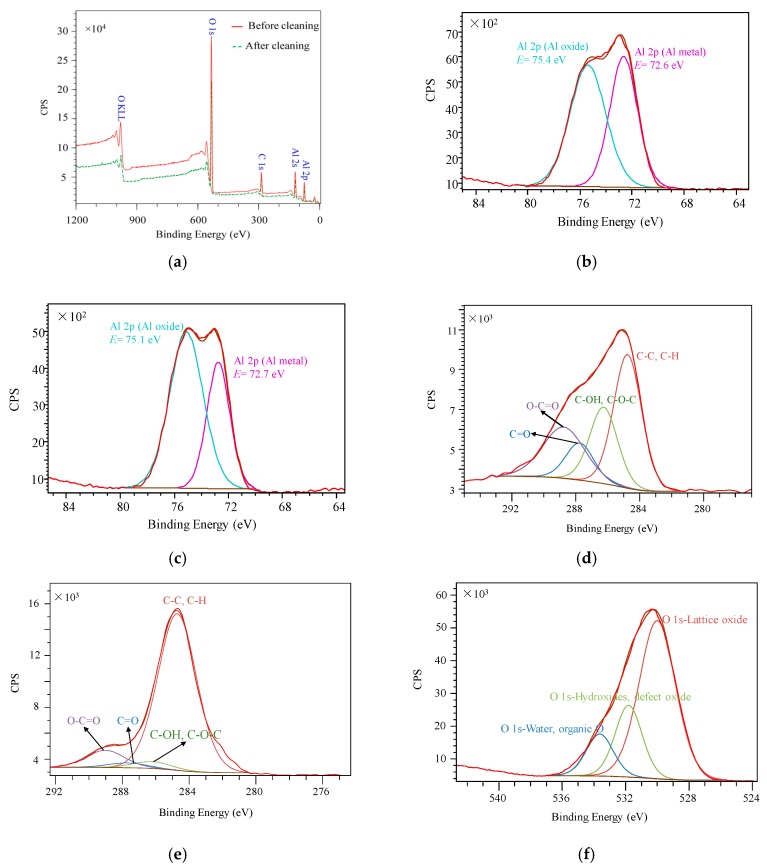

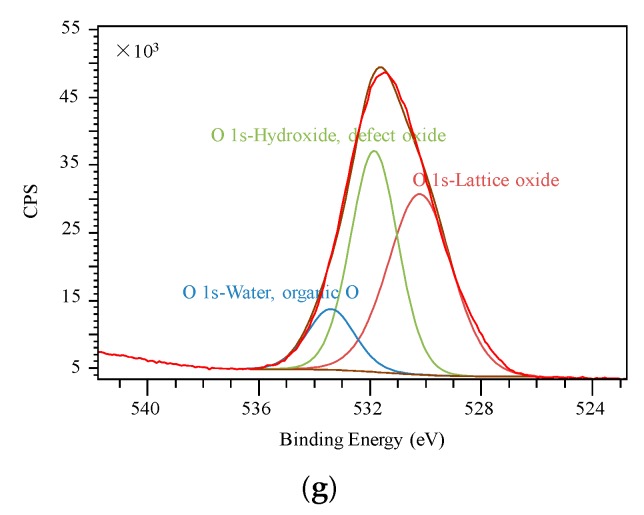
XPS spectra and curve fitting of laser treated sample #1. (**a**) The wide scan; (**b**) Al 2p peak of the uncleaned one; (**c**) Al 2p peak of the cleaned one; (**d)** C 1s peak of the uncleaned one; (**e**) C 1s peak of the cleaned one; (**f)** O 1s peak of the uncleaned one; (**g**) O 1s peak of the cleaned one.

The surface chemical state is one of the crucial factors affecting the SEY [26,27,28,29,30]. Therefore, XPS was carried out to ascertain the element concentrations of sample #1 before and after ultrasonic cleaning, as shown in Figure 5. 

Peak fitting was adopted using CasaXPS Version 2.3. The charging was found during the XPS analysis, which was compensated by shifting the adventitious carbon peak to 284.8 eV. Gaussian (30%)–Lorentzian (70%) peak shape was employed for the curve fitting of the Al 2p, C 1s, and O 1s peaks for uncleaned and cleaned laser processed aluminum alloy sample #1. The XPS wide scan of sample #1 identified the major constituents of aluminum, carbon, and oxygen elements. As shown in Table 2, compared to the aluminum and oxygen element concentrations of uncleaned sample #1, the concentrations of the cleaned ones decreased about 1.9% and 5.2%, respectively, while the concentration of the carbon element increased about 7.1%. 

In Figure 5a, the intensity drop in the XPS survey spectrum after cleaning may be related with the surface changes. As shown in Figure 5b,c, the concentrations of Al metal and Al oxide of sample #1 before and after ultrasonic cleaning were acquired by peak fittings. In Figure 5c, for cleaned laser treated sample #1, these two components, Al metal and Al oxide, were found at 72.7 eV and 75.1 eV, respectively, with the concentrations of 35.7% and 64.3%, respectively [31,32]. Before ultrasonic cleaning, the concentrations of Al metal and Al oxide were 45.2% and 54.8%, respectively. This result indicates that the concentration of Al metal decreased by 9.5% and the concentration of Al oxide increased after cleaning.

An increase of the carbon content of about 7.1 at % after cleaning was observed, as indicated in Table 2. Quantification of the amounts of C–C/C–H (carbon), C–OH/C–O–C (alcohol etc.), C=O (ketones), and O–C=O (esters or acids) parts of uncleaned and cleaned sample #1 are shown in Figure 5d,e. These four fitting peaks of C–C/C–H, C–OH/C–O–C, C=O, and O–C=O were found at the binding energies of 284.8 (±0.1) eV [33], 286.3 (±0.1) eV [34], 287.8 (±0.1) eV [35], and 289.3 (±0.1) eV [36], respectively. In contrast to the C 1s spectrum of uncleaned sample #1, the concentration of the C–C/C–H part of the cleaned one increases about 43.2%, with a concentration decrease of C–OH/C–O–C, C–O, and O–C=O parts of nearly 9.9%, 8.8%, and 14.5% severally. It indicates that the carbon content increase after cleaning is mainly caused by the concentration increase of the C–C/C–H part. In other words, more adventitious carbon after ultrasonic cleaning contributes to the carbon content increase. This can be explained by the more sphere/spheroidicity-like grains formed during ultrasonic cleaning, which led to the increase of the surface areas. More adventitious carbons may be attached on the cleaned surface, finally contributing to the carbon content increase. Additionally, the distinct decrease of the O–C=O part after cleaning means that the esters or acids were partly removed during ultrasonic cleaning.

The uncleaned and cleaned sample #1 oxide surfaces were decomposed into lattice oxide, hydroxides/defect oxide, and water/organic O parts [37,38,39], as indicated in Figure 5f,g. The main peaks at 530.0 (±0.1) eV are due to lattice oxide bonds. The peaks at 531.8 (±0.1) eV are assigned to hydroxides/defect oxide bonds. The peaks at 533.5 (±0.1) eV can be attributed to water/organic O bonds. The concentration of lattice oxide and water-organic O parts decreased about 18.9% and 1.3%, respectively. The content of hydroxides/defect oxide part increased about 20.2% after ultrasonic cleaning. Considering the element concentrations of laser treated sample #1 exhibited in Table 2, the oxygen concentration decrease can be ascribed to the decrease of lattice oxide content after cleaning.

Peak fittings of the Al 2p spectra for laser-processed sample #1 show that the ultrasonic cleaning might induce the concentration decrease of Al metal and the increase of Al oxide. The SEY of Al oxide is higher than that of Al metal. Thus, the increase of Al oxide and the decrease of Al metal may contribute to the SEY increase of cleaned samples.

**Figure 6:**
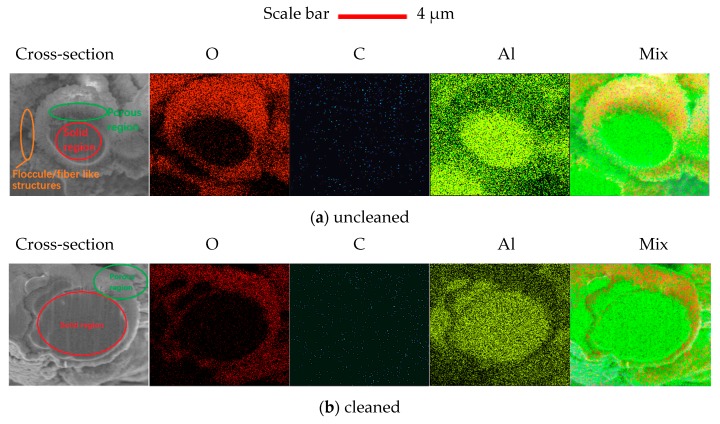
The morphologies and compositions of (**a**) uncleaned and (**b**) cleaned laser treated sample #1 were obtained by Scanning Electron Microscope and Energy Dispersion Spectrum.

The cross section morphologies and compositions of uncleaned and cleaned laser treated sample #1 are shown in Figure 6. The fracture surfaces were cut by a focused ion beam system. The porous and dense regions are labeled in Figure 6a,b. C element is nearly uniformly distributed on the porous and dense regions, due to the adventitious carbon adhesion on the cut and uncut surfaces. O content can be detected mainly from the floccule/fiber-like structures and porous region for cleaned and uncleaned surfaces. The density of the Al element in the dense region is much higher than that in the porous region. According to the XPS results above, the concentration of Al oxide in the floccule/fiber-like structures and porous region is higher than that in the dense region. This means aluminum alloy can react with active gases like oxygen in air during laser processing.

**Figure 7:**
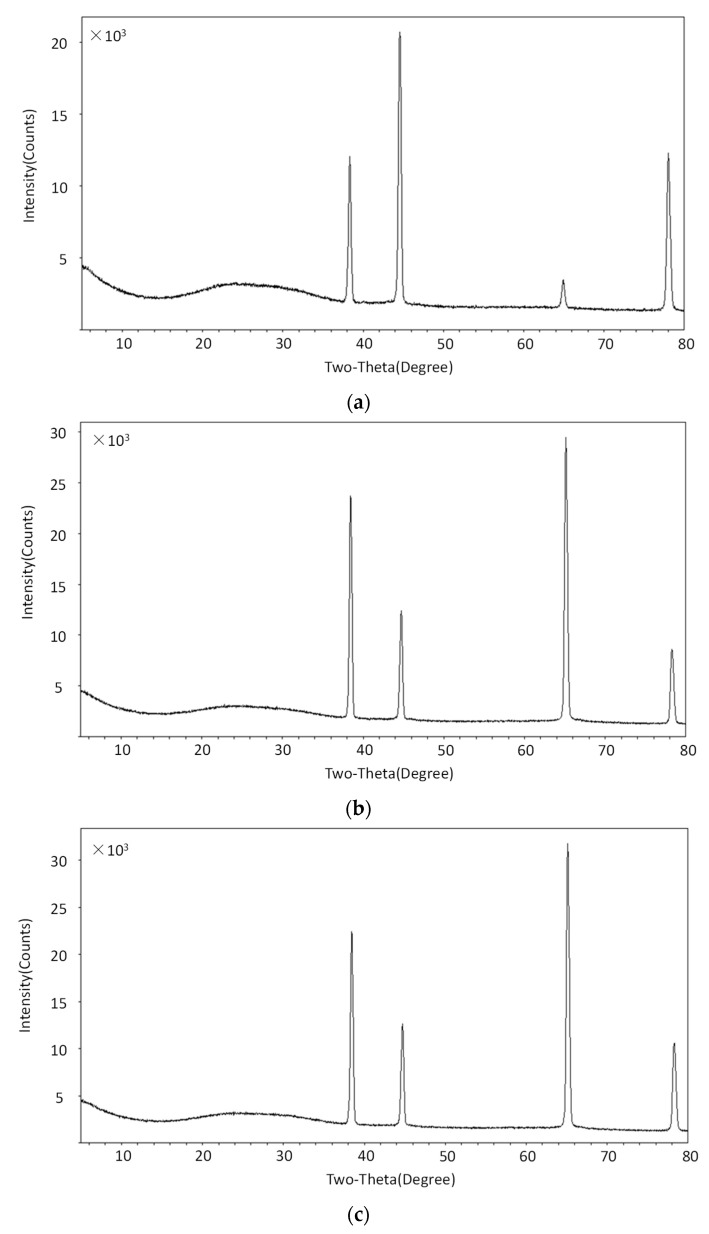
The XRD results of (**a**) untreated aluminum alloy, (**b**) laser treated aluminum alloy, and (**c**) cleaned laser treated aluminum alloy sample #1.

The XRD results of untreated aluminum alloy, laser treated aluminum alloy, and cleaned laser treated aluminum alloy sample #1 are shown in Figure 7. After laser processing, the positions of the Bragg reflection peaks were the same, while the half maximum intensity changed. The half maximum intensity variations are corresponding to the lattice microstrain [40]. It can be speculated that laser processing could induce the lattice microstrain of the aluminum alloy samples. The shapes of the laser treated aluminum alloy and cleaned laser treated aluminum alloy are basically the same. This indicates that the ultrasonic cleaning did not change the lattice parameters of the laser treated aluminum alloy sample by and large. 

The SEY increase after cleaning is mainly related to the change of surface morphology and surface chemical states. On the one hand, the SEY of metal oxide is higher than that of Al metals. From a theoretical point of view, the incident electrons energy is dissipated because of electron–electron collisions, and it excites less secondary electrons for metals [41]. The work functions of metals are higher than the electron affinity of insulators, and the energy loss in the conduction band of metals is higher than that of insulators. Thus, the moderate increase of Al oxide and the decrease of Al metal may lead to the SEY increase. Moreover, the introduction of some other impurities, such as adventitious carbon, may also result in the SEY increase. On the other hand, the floccule/fiber-like structures were removed after ultrasonically cleaning. Wang et al. [42] found that the fractal rectangle groove-like gap can decrease the SEY, while hemisphere-like structures can emit more secondary electrons. Thus, the surface morphology change may mainly give rise to SEY increase.

## 4. Conclusions

In summary, the effects of ultrasonic cleaning on the surface topography, surface chemistry, and SEYs of laser processed aluminum alloy samples were discussed and analyzed for the first time. The main points of this study were summarized as follows:

(1) Firstly, compared with the SEYs of uncleaned laser processed aluminum alloy samples, the SEYs of cleaned ones increase significantly. The ***δ***_max_ values of cleaned ones increase about 0.22–0.69. From the point of the application of laser processed aluminum alloy for electron cloud mitigation, the influence of ultrasonic cleaning should be considered, tested, and evaluated carefully in the future.

(2) Secondly, on the basis of the SEM results above, the removal of part of nano-sized floc/fibre-like floccule structures may be the main reason for the SEY increasing. On the one hand, the nano-sized floccule structures which could contribute to the capture of secondary electrons were mostly removed after cleaning. On the other hand, the sphere/stick-like volcanic sponges and curves formed by laser ablation could also result in the increase of secondary electrons emission. 

(3) Thirdly, with the increase of carbon content of around 7.1%, the aluminum and oxygen contents decreased about 1.91% and 5.2%, respectively, after cleaning. The Al content decreases slightly after cleaning, the concentration of Al metal decreased by 9.5%, and the concentration of Al oxide increased. The oxygen concentration decrease can be ascribed to the decrease of lattice oxide parts after cleaning. The peak fitting of C 1s curve indicates that the carbon content increase may be caused by the introduction of more adventitious carbon attached on the cleaned surface. Because more sphere/spheroidicity-like grains are formed after cleaning, the surface areas’ increase may induce more adventitious carbons attached on the cleaned laser treated surface.

(4) Fourthly, the XRD results indicate that laser processing could induce the lattice microstrain of the aluminum alloy samples. Meanwhile, the ultrasonic cleaning did not change the lattice parameters of laser treated aluminum alloy sample on the whole. 

(5) In short, considering the SEY increase after ultrasonic cleaning, the cleaning time should be reduced accordingly. Other cleaning methods or the combination with other methods should be also considered in the future, such as glow discharge.

## Figures and Tables

**Table 1:** Laser parameters for aluminum alloy samples.

Sample	Pitch Spacing/μm	Scanning Speed/mm s^–1^	Before Ultrasonic Cleaning			After Ultrasonic Cleaning		
			*δ*_max_	*E*_max_^1^/eV	*Ra*/μm	*δ*_max_	*E*_max_/eV	*Ra*/μm
#1	15	100	0.99	3000	10.7	1.43	2700	9.4
#2	20	150	1.05	2400	7.5	1.74	2600	6.9
#3	5–25	150	1.16	3000	14.5	1.38	400	12.9

^1^*E*_max_ is the maximum SEY within the measured range.

**Table 2:** Quantification of the concentrations of the laser processed aluminum alloy sample #1 before and after ultrasonic cleaning by XPS analysis (at %).

Conditions	Al/at %	C/at %	O/at %
Before cleaning	29.3%	20.1%	50.6%
After cleaning	27.4%	27.2%	45.4%
